# Developing a digital health strategy for people who use drugs: Lessons from COVID-19

**DOI:** 10.1177/20552076211028404

**Published:** 2021-06-28

**Authors:** Melissa Perri, Adrian Guta, Marilou Gagnon, Matt Bonn, Pamela Leece, Ahmed M Bayoumi, Nanky Rai, Natasha Touesnard, Carol Strike

**Affiliations:** 1 Dalla Lana School of Public Health, University of Toronto, Toronto, Canada; 2 MAP Center for Urban Health Solutions, St. Michael’s Hospital, Toronto, Canada; 3 School of Social Work, University of Windsor, Windsor, Canada; 4 Canadian Institute for Substance Use Research, Victoria, Canada; 5 Canadian Association of People Who Use Drugs, Dartmouth, Canada; 6 Canadian Students for Sensible Drug Policy, Ottawa, Canada; 7 Public Health Ontario, Toronto, Canada; 8 Department of Family and Community Medicine, University of Toronto Faculty of Medicine, Toronto, Canada; 9 Institute of Health Policy, Management and Evaluation, University of Toronto, Toronto, Canada

**Keywords:** Harm reduction, COVID-19, digital health, overdose prevention, pandemic

## Abstract

COVID-19 has significantly exacerbated negative health and social outcomes for people who use drugs (PWUD) around the world. The closure of harm reduction services, ongoing barriers to employment and housing, and pre-existing physical and mental health conditions have increased harms for diverse communities of PWUD. Adapting current models of health and human service delivery to better meet the needs of PWUD is essential in minimizing not only COVID-19 but also drug-related morbidity and mortality. This article draws on research, practice, and advocacy experiences, and discusses the potential for digital health tools such as remote monitoring and telecare to improve the continuum of care for PWUD. We call for a digital health strategy for PWUD and provide recommendations for future program development and implementation.

## Introduction

The severe acute respiratory syndrome coronavirus 2 (SARS-CoV-2) (COVID-19) pandemic has necessitated a digital health care revolution (e.g., increased use of telecare, telehealth, telemedicine, virtual care, technology-enabled care, technology-driven interventions) due to related public health physical distancing requirements to reduce transmission.^1,2^ Many logistical barriers to the scale-up of digital care that previously seemed insurmountable have been overcome in the context of this global emergency.^2,3^ The COVID-19 pandemic has been associated with a spike in substance use, relapse, and overdoses which has served to worsen the overdose epidemic across North America.^4^ To better support people who use drugs (PWUD) during this especially difficult time, many healthcare providers have turned to established and emerging technologies to engage PWUD, including to provide opioid agonist therapy (OAT), follow-up care, consultation, and counseling.^3^ However, pre-existing barriers to accessing health and human services faced by marginalized communities are likely to play an important role in determining who benefits from this turn to digital care, particularly for PWUD. Lessons gathered from the first wave of COVID-19 demonstrate a need for the adaptation of current service delivery measures to better meet the unique needs of PWUD. In this article, we consider the impacts of COVID-19 on harm reduction programing and the possibility of developing a digital health strategy to prevent treatment interruptions and enable expanded care options. This article is based on the team’s respective experience as substance use and harm reduction researchers, health and human service providers (medicine, nursing, social work), activists and persons with lived experience. We have integrated literature and insights from a program of research on the needs of PWUD during the pandemic, clinical practice experience, and lessons from our networks.

## What are the health impacts of COVID-19 on people who use drugs?

The COVID-19 pandemic has significantly exacerbated health disparities for PWUD worldwide due to disruptions in drug supplies, drug markets, and access to harm reduction care.^5,6^ Increasing contamination and toxicity of the illicit drug supply has been reported by PWUD and drug checking services in Canada and the United Kingdom and is contributing to worsening overdose rates.^7,8^ Factors that limited access to health and human services prior to the pandemic include stigma and discrimination, poverty, structural racism and the criminalization of drug use.^9,10^ PWUD may be at risk for COVID-19 related complications due to a combination of biopsychosocial factors such as income, housing, and food insecurity which reduce one’s ability to isolate, substance use related risks (e.g., communal use and smoking drugs), and high rates of comorbid illnesses (e.g., unsuppressed HIV infection and chronic hepatitis C infection (HCV)).^8,11^ PWUD may be at risk of COVID-19 transmission through sharing supplies relating to smoking equipment, which has been documented to be more common than sharing injection supplies.^9^ Additionally, PWUD have experienced increasing opioid-related morbidity and mortality during the pandemic.^12^

Despite being known for its social welfare system and relatively liberal approach to the management of illicit drug use relative to the United States, Canada has had concerning trends of opioid-related deaths since the onset of the pandemic. In the province of British Columbia, opioid overdose deaths in November 2020 (n = 153) increased by 89% compared to November 2019.^13^ Ontario, another Canadian province, reported similar trends, with approximately 55 people dying every week of suspected overdoses, roughly a 25% increase in overdose deaths from the median in 2019.^14^ This represents the highest opioid overdose death rate since monitoring began.^15^ Similar findings have been reported across the United States where reports indicate that there were over 81,230 drug-related deaths in the year leading up to May 2020, with the highest percentages occurring between March 2020 and May 2020 as a result of COVID-19.^16^ Experiences with drug and COVID-19 related morbidities, mirrored with the lack of available services has left many PWUD relying on emergency rooms for care.^17^ This lack of availability has escalated drug-related harms internationally, with reports from contexts as diverse as Bangalore and the United Kingdom indicating an increase in alcohol and substance-related withdrawal symptoms.^18,19^

## What challenges are faced by addictions and harm reduction programing as a result of COVID-19?

Harm reduction programs, many of which are precariously funded in the best of times, have faced barriers to maintaining adequate service levels during COVID-19. This has included increased physical distancing and infection control requirements (e.g., fewer clients served and more time between clients), a lack of personal protextive equipment and infection control, infections among staff and clients which has led to temporary closures and service restrictions, and staffing and financial resources being redeployed to other areas of public health.^11^ Many addiction treatment programs have experienced similar barriers, along with concerns about physician availability, and some have reduced new client intakes and closed treatment programs (e.g. day programs) which reduces the availability of OAT.^19^ Many PWUD have experienced problems in accessing sterile equipment, overdose prevention education, and community support. A further consequence of program closures are reduced opportunities for peer employment and engagement, disconnecting PWUD from their communities and reducing well-being.^6,9.10^ To develop accessible and effective harm reduction interventions, the unique challenges faced by both service users and providers because of COVID-19 must be considered.

Key lessons from the first wave of COVID-19 in Canada, and subsequent localized lockdowns, include the need for accessible and scaled-up OAT, safe supply programs, increased drug checking, and the importance of maintaining the continuity of care for PWUD (inclusive of HIV and HCV care cascades).^20,21^ An innovative approach was launched in British Columbia which loosened prescribing guidelines as part of a broad risk mitigation strategy to support PWUD to physically distance by providing them with a prescription for oral hydromorphone and benzodiazepines and some stimulants.^10^ There have been calls for the scale-up of a ‘safe supply’ (prescription of safe drugs to offset the risk of illicit street drugs and meet the needs of PWUD), but questions remain about access.^22^ Incorporating telemedicine and remote care strategies in both prescribing practices and delivery of care to PWUD is an effective alternative to support existing clients and patients, minimize stigma associated with in person care for PWUD (i.e., client’s experiencing stigma as a result of being seen accessing harm reduction or addiction treatment services), improve willingness to participate in care (e.g., with the avoidance of factors such as urine screening), and expand the availability of care to remote or rural regions.^23,24^

## What role do digital strategies play in providing care to people who use drugs?

***Using existing telecare technology to meet the needs of PWUD***: In order to provide care for PWUD during the COVID-19 pandemic, some physicians and pharmacists have used telehealth options (phone and video consultation) to offer OAT and ‘safe supply’ prescribing.^25–28^ The integration of telecare for providing such treatments have been recommended by the Canadian Research Initiative in Substance Misuse.^26^ Telecare is also being used in the management of acute withdrawal symptoms and the British Columbia Centre on Substance Use has outlined recommendations for physicians to treat alcohol withdrawal symptoms. When deemed safe, care for withdrawal may be provided through telecare services, such as conducting regular telephone or video assessments for clients.^27^ This has allowed PWUD and healthcare providers to maintain COVID-19 physical distancing protocols while maintaining treatment regimens and connection, and minimizing the risk of alcohol or drug-related morbidity and mortality.^28^ As telecare develops, team-based care options inclusive of physicians, nurse practitioners, social workers, pharmacists, and harm reduction workers with lived/living expertise of drug use should be offered to PWUD to ensure their psychological and material needs are also met.

Despite the advantages telecare presents for PWUD, the challenges faced in developing and implementing these programs are significant. Common barriers include the lack of acceptance or limited availability of technical resources for prescribers or patients.^3^ Many people living in poverty do not have basic phone coverage to make or receive a telehealth call let alone the data to videoconference with a provider.^11^ Bruneau and colleagues^26^ identified the following factors which physicians must consider when adapting the provision of drug-related care to telecare options: the accessibility of telecare options must be assessed on a case-by-case basis, and platforms used for telecare implementation must be secure and should be explained prior to every consultation to ensure informed consent. Advancing telecare should include discussions about access and in-person options. In the meantime, we are aware of health and human services which have remained open serving as brokers to assist PWUD to access care providers and make and receive phone and video calls. Before the pandemic, many harm reduction programs offered a critical service by letting PWUD access Wi-Fi networks. With other services like public libraries and coffee shops closed, this basic service is more important than ever.

***Beyond telecare to complement the care of PWUD with digital options*:** In addition to telecare, a range of digital health applications and services have implications for PWUD. The availability of apps and other technology platforms to support safer drug use are limited, but comparable models are available to support managed alcohol consumption and smoking cessation.^29^ Technology-based tools or apps are typically used to promote behaviour change and mitigate risk behaviours among PWUD. However we can draw upon tools that currently exist, and some under development, to support PWUD to use drugs safely and reduce the risk of overdose.^30^ For example, the Overdose Risk InfOrmatioN tool (ORION) is an innovative e-health tool implemented in clinical settings across Europe, which incorporates overdose risk assessments and provides individuals with information on future harm reduction practices and behaviours which aim at reducing overdose rates (i.e. testing drugs prior to use).^31^ An evaluation of ORION (n = 194) demonstrated that over half of the individuals who engaged with the tool developed some sense of increased overdose prevention education.^31^

Several examples demonstrate how online services can be used to provide individuals with addiction treatment.^32,33^ However, many PWUD experience barriers to engaging with treatments programs (i.e., ineligibility for addiction services due to abstinence protocols or waitlists due to limited capacity).^32,33^ Alternative approaches need to be considered for persons who are not ready or interested in treatment and abstinence. To this end, mobile apps are being developed and implemented to assist in providing remote care for PWUD.^34^ Apps aimed at minimizing drug-related harms include services such as remote supervision (e.g., being linked to someone who knows that the app user is about to take drugs and can send assistance if they believe an overdose is happening) and guidance about safer drug use and overdose prevention.^34^ The use of digital services and apps must also be considered in relation to naloxone distribution. Services which foster delivery of naloxone kits have been developed^35^ and should be integrated within regularly provided digital care for PWUD to minimize risks of overdose as a result of using alone. In order for the effective distribution of naloxone through digital methods, the various distribution policies between jurisdictions must be consolidated.^36^ Additional features of harm reduction based apps include information on social services available to individuals or a wearable component (e.g., a smart watch) that detexts lack of movement associated with overdoses.^34^ The integration of mobile apps for the delivery of health and human services may also improve the development of relationships between PWUD and service providers, and mitigate fear, distrust, or discomfort associated with in person care.

In light of the COVID-19 pandemic, British Columbia has implemented the Lifeguard App, which allows PWUD to communicate with emergency services while they are using substances to minimize risk of fatal overdoses.^37^ The initiative is a partnership between the Provincial Health Services Authority, regional health authorities and Lifeguard Digital Health.^37^ The COVID-19 pandemic has highlighted urban/rural health disparities and created opportunities to assess such supports for PWUD in rural and remote areas. The COVID-19 pandemic has furthered policy discussions about the digital divide^38,39^ (e.g., disparities between students who can stream and actively participate in on-line education and the requirement to have a smart phone to book an appointment at a COVID-19 vaccine clinic) and how to scale-up broadband coverage in underserved communities. As well, whilst we recognize the life-saving potential of these apps, and that public private partnerships may speed up their development,^40^ we call for these technologies to be user controlled and that data not be shared with law enforcement.^41^ The need to ensure privacy of personal health information is essential in minimizing the utilization of data by private corporations involved in the development of technologies.

## How do we develop a sustainable digital health strategy for people who use drugs?

Despite the need to simultaneously overcome long-term disparities and deliver rapid implementation and scale-up during COVID-19, the use of technology mediated care is promising for PWUD. However, this digital turn requires additional consideration for those who do not have access to health, social, and harm reduction services, particularly PWUD who reside in remote or rural regions and who lack access to supervised consumption sites, needle and syringe distribution programs and other common harm reduction practices.^42^ Previous work has identified the potential advantage that incorporating technology has for PWUD, such as allowing for personalized care and cost effective ways to reach a wide variety of individuals.^43^ Regions such as Australia have developed a nationwide telecare strategy, which focuses on delivering guidance to health care providers on how to integrate telecare in their respective practices (e.g., provides hands on training with technological strategies).^44^ The Australian example has important implications for Canada and the United States, but differences in how health and public goods are funded remain important considerations. As noted previously, despite Canada having universal access to healthcare, considerable barriers and disparities exist between rural and urban settings and among those without health insurance due to housing insecurity or precarious immigration status. In the United States, access to healthcare with considerable variation between urban/rural and on a state-by-state basis, and draconian drug-polices will need to be considered and overcome. Both countries have ongoing racial disparities with Black, Indigenous, and people of color, including those who use drugs, experiencing stigma, marginalization, and exclusion in the healthcare system. Sustainability plans should be developed with equitable co-leadership from directly impacted communities of PWUD, especially those facing intersecting barriers to accessing healthcare as a result of structural violence and those facing higher rates of criminalization due to systemic anti-Indigenous and anti-Black racism.

The path towards scale-up and sustainability will require research in, and evaluation of, the development of technology-based interventions for PWUD. A factor that must be considered in the scaling-up of technology-based interventions is the low-barrier nature of harm reduction programs. This may result in many interventions offering a wide variety of technology (i.e. smart phone or Wi-Fi), considering how the COVID-19 response has limited access to Wi-Fi and other technology for PWUD, and ensuring that the lack of continuous access to technology is not used to discharge or dismiss clients. To effectively implement digital care for PWUD, research must be done with members of the community and be attentive to potential ethical, legal, and safety concerns. Above increased attention to research and program development, we call for a systems level change with integration of strong legal protextions for PWUD and a approach led by and for PWUD.

## Conclusion

COVID-19 has exacerbated existing inequities among PWUD while creating unique challenges for healthcare providers working with PWUD. To effectively develop and provide health care to PWUD during and after the COVID-19 pandemic, primary care, mental health, addiction, and harm reduction services should continue to integrate and advance digital health care strategies. As the overdose crisis continues to merge with the COVID-19 pandemic we need to utilize all available methods to reduce drug related harms and overdose fatalities. Scale up of these services is necessary now and long past the containment of COVID-19 as a strategy for addressing the decades long overdose crisis. Meaningful involvement of those with lived and living expertise of drug use is needed to ensure that these strategies do not increase harm.
